# Diagnostic Considerations for Non-*Acanthamoeba* Amoebic Keratitis and Clinical Outcomes

**DOI:** 10.3390/pathogens11020219

**Published:** 2022-02-08

**Authors:** Siobhan Moran, Ronnie Mooney, Fiona L. Henriquez

**Affiliations:** School of Health and Life Sciences, University of West Scotland, Stephenson Place, Glasgow G72 0LH, Lanarkshire, UK; B00265235@studentmail.uws.ac.uk (S.M.); fiona.henriquez@uws.ac.uk (F.L.H.)

**Keywords:** keratitis, amoeba, hartmannella, acanthamoeba, vannella, vahlkampfia, vermamoeba, diagnosis, pathogenesis

## Abstract

Cases of amoebic keratitis involving species other than *Acanthamoeba* are hypothesised to be underdiagnosed and poorly understood. Amoebic keratitis is debilitating and associated with chronic visual impairment. Understanding associated symptoms of non-*Acanthamoeba* amoebic keratitis could facilitate new diagnostic procedures and enable prompt treatment, ultimately leading to improved patient outcomes. Thus, a review of the literature was undertaken surrounding non-*Acanthamoeba* amoebic keratitis. Cases were geographically widespread and mostly confined to contact lens wearers ≤ 30 years old exposed to contaminated water sources and/or demonstrating poor lens hygiene. *Vermamoeba vermiformis* (previously *Hartmanella vermiformis*) was the most common causative agent, and a moderate number of mixed keratitis cases were also reported. A crucial disease indicator was early onset stromal deterioration/ulcerations, reported in 10 of the studies, usually only occurring in advanced *Acanthamoeba* keratitis. Mixed infections were the most difficult to treat, often requiring keratoplasty after unsuccessful combination treatment regimens. New diagnostic measures for non-*Acanthamoeba* amoebic keratitis should consider early onset stromal disease as a key disease indicator. Deep corneal scrapes are also necessary for accurate amoebic identification. Moreover, a combination approach to diagnosis is advised and should involve culture, microscopy and PCR techniques. In vitro drug sensitivity tests should also be conducted to help develop patient-specific treatment regimes.

## 1. Introduction

Amoebic keratitis is of growing clinical concern given the recent rise in cases globally and the difficulties associated with accurate diagnosis and treatment [1]. A recent outbreak of amoebic keratitis has been described in the UK, with other developed countries following similar trends [1,2,3,4,5]. *Acanthamoeba* spp. are most commonly associated with amoebic keratitis, and most cases can be linked to contact lens wear and poor contact lens hygiene [6,7,8]. Although not the only risk pathways of infection, exposure of contact lenses to contaminated water, overwearing lenses and improper cleaning and storage of lenses and cases (e.g., using tap water for cleaning purposes) enable amoebic adhesion and growth on lens surfaces and subsequently increase the risk of corneal infection [7].

While *Acanthamoeba* keratitis is the most common form of amoebic keratitis and as such has been the subject of extensive research, there are other potential contributing organisms that are less considered [1]. Despite a lack of conclusive evidence demonstrating that amoebae, with the exception of *Acanthamoeba*, are capable of causing keratitis in isolation, there have been several reported cases of positively identified non-*Acanthamoeba* amoeba species during keratitis infections documented in the literature. For example, *Vermamoeba vermiformis* (previously *Hartmannella vermiformis*), *Vannella* and *Vahlkampfia* species have all been associated with amoebic keratitis, and a lack of knowledge surrounding their potential for infections may hinder effective diagnostic or treatment protocols [9].

Because of the poorly understood role of non-*Acanthamoeba* amoebic keratitis, we have conducted an extensive review of the literature to further detail its prevalence, diagnoses, disease symptoms and treatments. In doing this, we aim to further the accuracy of diagnostic procedures that might aid in the rapid diagnosis and treatment of these pathogens.

## 2. Results

**Figure 1:**
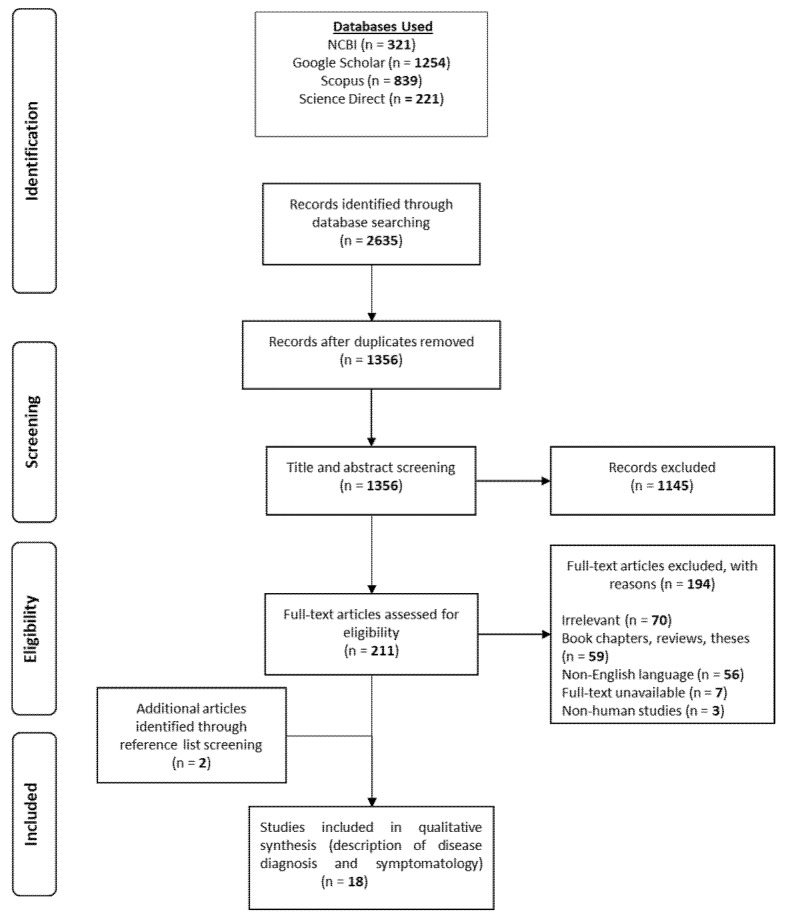
PRISMA flow diagram.

A total of 18 articles satisfied the inclusion guidelines set for this review (Figure 1), and key information was extracted from each article (Table 1). Studies were conducted in various locations worldwide, and study participants were diverse with respect to gender, age and history of contact lens wear (Table 2). Of all the identified case studies, participant specifics were only detailed in the study conducted by Hajialilo et al. [10]; consequently, they are excluded from Table 2’s participant section. Cases were described more frequently in females with 61.5% (8 female to 5 male) and also more frequent in participants within the 30-and-under age category (69.2% of cases, 9 ≤ 30 y/o to 4 > 30 y/o). Cases were much more apparent in contact lens wearers than in non-contact lens wearers, with 92% of cases linked to contact lens wear (61 to 5).

Of all infections, 21% were identified in conjunction with an additional amoebic infection, with half being a co-infection with an *Acanthamoeba* spp. Isolated infections with *Vermamoeba vermiformis* were most frequent, followed by isolated *Vahlkampfia* infections (53% and 23%, respectively). It should also be noted that in many cases, non-amoebic co-infective agents were identified during diagnoses, and in others, evidence of extensive screening to exclude the presence of additional microbial or viral pathogens was not provided. Thus, the role of these amoebae as causative agents of keratitis and not as vectors for the transmission of other microbial pathogens, as has been described in other free-living amoebae [27], remains inconclusive. Nonetheless, the most common clinical manifestations of non-*Acanthamoeba* amoebic keratitis included pain (61% of studies), inflammation and irritation (61%), stromal keratitis and ulcerations (56%), epithelial abnormalities (50%), ring infiltrates (50%), impaired vision (44%) and photophobia (44%) (Table 3).

**Figure 2:**
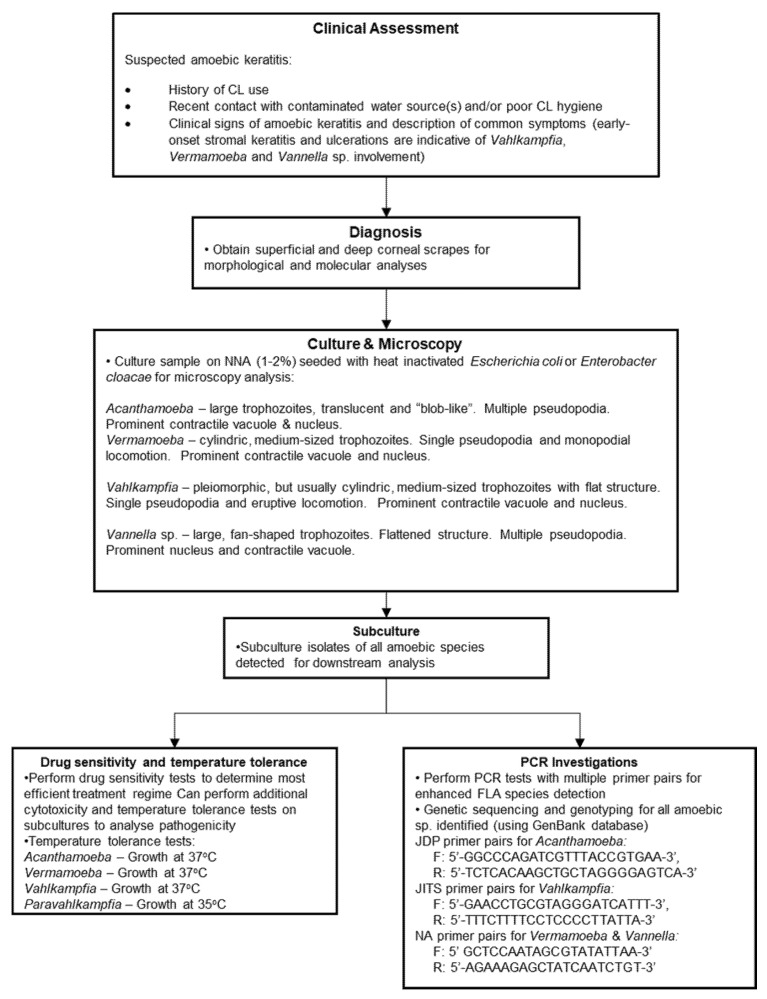
Diagnostic flowchart for non-*Acanthamoeba* amoebic keratitis. Stromal involvement is a key disease indicator. Deep corneal scrapes can facilitate non-*Acanthamoeba* amoebic detection, and the use of multiple primer pairs can aid detection of multi-amoeba sp. in a sample.

Diagnostic techniques vary between studies, with earlier studies relying primarily on culture and microscopy techniques to identify suspected pathogens. More recent diagnoses, however, have involved the use of 18s rRNA sequencing techniques to provide more accurate diagnoses with genus-specific primers (Figure 2). Additionally, temperature tolerance tests have been performed, as they are deemed to be good indicators of pathogenic virulence [26,28].

Therapeutic approaches were broad and predominantly included drug combinations for all forms of amoebic keratitis (Table 1). This approach is similar to the approach taken with AK because no single anti-amoebic agent exists that can successfully eradicate all *Acanthamoeba* genotypes; hence, drug combinations are used [7,29]. Medical interventions included anti-amoebic agents (topical biguanides and aromatic diamidines), antibiotics, antifungals, antivirals and anti-inflammatory medications. Notably, several studies reported the need for keratoplasty due to poor treatment responses (Table 1) [12,13,14,17,18,22,24,26]. This emphasises the difficulty of finding an effective therapeutic agent to cure amoebic keratitis and highlights the possibility of drug resistance in other FLA besides *Acanthamoeba.*

## 3. Discussion

This review demonstrates that cases of non-*Acanthamoeba* amoebic keratitis are occurring and are geographically widespread; no single location showed a significantly higher incidence rate than any other (Table 2). Notably, however, disease incidence appears to be confined to developed countries, thus following a similar pattern to AK occurrences [7]. Although it is unclear, this might be the result of differences in diagnostic techniques between countries. In terms of patient demographics, Table 2 shows that the majority of individuals diagnosed with non-*Acanthamoeba* amoebic keratitis were ≤30 years old, and a significantly high proportion of positive cases were associated with contact lens wearers (61 cases vs. 5 non-CL wearers who had suffered ocular trauma). These findings also coincide with typical AK observations in which amoebic keratitis predominantly occurs in young, economically active adults who are most likely to wear contact lenses [2,7].

The similarities to AK infections complicate diagnoses. Generally, treatment outcomes of AK infections coincide with the speed by which accurate diagnoses can be made [7,30]; thus, a similar emphasis should be placed on rapid diagnosis in non-*Acanthamoeba* amoebic keratitis. Differentiation between amoebic infections is difficult; despite this, early onset stromal keratitis and ulcerations are indicative of *Vahlkampfia*, *Vermamoeba* and *Vannella* sp. involvement and could be useful during early observations (see Table 1) [9,11,13,14,15,17,19,20,22,24]. While microscopic and culture techniques can be applied during diagnostics, species identification using PCR tests remains the most accurate strategy for diagnosis and should be utilised at the earliest possible convenience [1]. We provide a more comprehensive diagnostic workflow for suspected amoebic keratitis in Figure 2. The presence of co-infectious agents in amoebic keratitis is common, and several non-amoebic species were also identified that can complicate treatment, diagnosis and the overall understanding of amoebic influence during infections [11,12,17,18]. For example, Kennedy et al. (1995) [11] reported growth of *Staphylococcus aureus* from corneal swabs, while Aitken et al. (1996) [12] noted the presence of yeast-like fungi on the corneal surface of the patient. Advances in molecular techniques allow more comprehensive screening for co-infectious agents and should be conducted in tandem with a traditional culture-based approach to assess the most appropriate course of action for the patient.

Despite the rarity of non-*Acanthamoeba* amoebic keratitis, the literature compiled within this review demonstrates that care must be applied during the initial diagnoses of amoebic keratitis to ensure the most suitable treatment method is applied. It is also worth noting the recent rise in AK infections globally [2,3,4,5] and that consideration must also be given as to whether non-*Acanthamoeba* causative agents of amoebic keratitis follow a similar trend. Overall, we demonstrate here that non-*Acanthamoeba* amoebic keratitis follows a similar pattern with regard to patient demographics and treatment regimens and that infections generally coincide with contact lens wear. We also highlight differences in disease presentation and methods of diagnoses. Despite its low prevalence, a broad-spectrum approach should be applied to the initial treatment of amoebic keratitis, which will necessitate further investigation into how AK therapies interact with other causative agents of amoebic keratitis. Furthermore, the role of non-*Acanthamoeba* amoebic species in keratitis infections has yet to be fully elucidated and, therefore, warrants additional research.

## 4. Materials and Methods

Four key databases were accessed: Scopus, Science Direct, Google Scholar and The National Center for Biotechnology Information (NCBI). The search criteria narrowly focused on *Vahlkampfia*, *Vermamoeba (Hartmannella)* and *Vannella* sp. and their involvement in keratitis. No date restrictions were applied due to the elusive nature of non-*Acanthamoeba* amoebic infections and the scarcity of relevant literature. Key words combined with Boolean operators and nested search strings were used to ensure results were confined to the topic of interest. Figure 1 details the flow path by which studies of non-*Acanthamoeba* amoebic keratitis infections were identified. All reported cases of keratitis associated with *Vahlkampfia* and *Vermamoeba vermiformis* (*Hartmannella*), coupled with descriptions of symptomatology and diagnostic measures, were included in this review. No restrictions to publication date, country, patient gender, race or age were applied. Studies selected for inclusion in this review were analysed against a Scottish Intercollegiate Guidelines Network flowchart [31], as recommended by the University of Strathclyde [32], to accurately determine study type. All studies were established to be case reports (CR) or case series (CS). Thereafter, each paper was quality-assessed against two critical appraisal tools provided by The Joanna Briggs Institute [33] and the Centre for Evidence-Based Management [34]. Data were presented as both total counts and percentages of overall data.

## Figures and Tables

**Table 1:** Suspected or confirmed cases of non-*Acanthamoeba* amoebic keratitis (1995–2019).

First Author, Year and Journal	Study Type and Location	Patient Information and Research Aim	Clinical Manifestations	Laboratory Investigation(s)	Findings	Treatment
Kennedy et al. (1995) *The lancet* [11]	CR (Ireland)	Analysing causative agent(s) of keratitis in a 17-year-old F soft CL wearer	Corneal erosion and stromal ring infiltration	Corneal scrapes: culture and microscopy	*Vermamoeba* detected (in isolation)	Polyhexamethylene
Aitken et al. (1996) *Ophthalmol* [12]	CR (UK)	Analysing causative agent(s) of keratitis in a 21-year-old M soft CL wearer	Eye pain, watery discharge, corneal lesion, inflammation, epithelial defect, oedema, decreased visual acuity, photophobia, keratic precipitates and endothelial damage	Corneal scrapes and corneal biopsy: culture and microscopy	*Vermamoeba* and *Vahlkampfia* detected	Propamidine isethionate, neomycin sulfate, prednisolone, atropine and keratoplasty
Inoue et al. (1998) *American Journal of Ophthalmol* [13]	CR (Japan)	Analysing causative agent(s) of keratitis in a 54-year-old F hard CL wearer	Eye pain, foreign body sensation, stromal ring infiltration, stromal ulcer, corneal lesion, impaired vision, epithelial defect, photophobia and anterior chamber inflammation	Corneal scrapes: culture and microscopy	*Acanthamoeba* and *Vermamoeba* detected	miconazole, fluconazole, natamycin, ofloxacin and keratoplasty
Aimard et al. (1998) *Clinical Infectious Diseases* [14]	CR (France)	Analysing causative agent(s) of keratitis in a 40-year-old F CL wearer	Stromal involvement observed and symptoms described as “typical of *Acanthamoeba*“	Corneal scrapes and corneal biopsy: culture and microscopy	*Acanthamoeba* and *Vermamoeba* detected	Neomycin, polymyxin, fluconazole, hexamidine, propamidine and keratoplasty
Alexandrakis et al. (1998) *Arch Ophthalmol* [15]	CR (USA)	Analysing causative agent(s) of keratitis in a 30-year-old M non-CL wearer who incurred ocular trauma	Severe ocular pain, irritation, stromal infiltrates, corneal oedema, epithelial defect and inflammation	Corneal scrapes: culture and microscopy	*Vahlkampfia* detected (in isolation)	Propamidine, polyhexamethylene, neomycin, polymyxin, bacitracin zinc and Clotrimazole
Bennett et al. (1998) *The British Journal of Ophthalmol* [16]	CS (UK)	Analysing causative agents of keratitis in a small cohort (24 M and 31 F patients with presumed microbial keratitis)	Central or peripheral infiltrates	Corneal scrapes: culture and microscopy	1 *Vahlkampfia* positive case (detected in isolation in CL wearer)	Gentamicin and Cefuroxime
Michel et al. (2000) *Parasitology Resources* [17]	CR (Germany)	Analysing causative agent(s) of keratitis in a 24-year-old F CL wearer	Pain, photophobia, reduced visual acuity, inflammation, corneal ulcer, conjunctival hyperaemia and central ring infiltrate	Corneal and CL swabs: culture and microscopy	*P. aeruginosa, Vermamoeba* and two *Vannella* sp. detected	Gentamicin, cefazolin, propamidine, artificial tears and keratoplasty
Scheid (2007) *Parasitology Resources* [18]	CR (Germany)	Analysing causative agent(s) of keratitis in a 24-year-old F CL wearer	Inflammation, impaired vision, photophobia, central ring infiltrate and severe pain	Corneal and CL swabs: culture and microscopy	*P. aeruginosa, Vermamoeba* and two *Vannella* sp. detected	Gentamicin, cefazolin, propamidine artificial tears and keratoplasty
Lorenzo-Morales et al. (2007) *Parasitology Resources* [19]	CR (Spain)	Analysing causative agent(s) of keratitis in a 21-year-old M soft CL wearer	Severe pain, reduced visual acuity, photophobia, inflammation and stromal keratitis	Corneal scrapes: culture and microscopy; PCR tests	*Acanthamoeba* and *Vermamoeba* detected	Tobramycin, Propamidine, povidone-iodine, diclofenac and ofloxacin
Yera et al. (2008) *The British Journal of Ophthalmol* [20]	CS (France)	Analysing causative agent(s) of keratitis in a small cohort (37 M and F patients with suspected AK). All CL wearers	Pain and stromal ring infiltrates	Corneal scrapes: culture and microscopy; CL and CL case investigations; PCR tests	1 *Vermamoeba* positive case (detected in isolation) 1 *Vahlkampfia* positive case (detected in isolation)	Unexplained
Ozkoc et al. (2008) *Journal of Medical Microbiology* [21]	CR (Turkey)	Analysing causative agent(s) of keratitis in a 61-year-old M non-CL wearer who incurred ocular trauma	Irritation, pain, redness, reduced visual acuity, corneal oedema, epithelial defect and epithelial erosions	Corneal scrapes: culture and microscopy; PCR tests	*Paravahlkampfia* and herpes simplex virus detected	Acyclovir, propamidine and polyhexamethylene
Niyyati et al. (2010) *Experimental Parasitology* [22]	CR (Iran)	Analysing causative agent(s) of keratitis in a 35-year-old F soft CL wearer	Severe pain, redness, irritation, ulceration, photophobia and opacity in the left eye	Corneal scrapes: culture and microscopy; PCR tests; CL and CL case investigations	*Acanthamoeba* and *Vahlkampfia* detected	Propamidine and keratoplasty
Mattana et al. (2012) *Mappe Parassitologiche* [23]	CS (Italy)	Analysing causative agent(s) of keratitis in a small cohort with suspected early stage *Acanthamoeba* keratitis	Diffuse punctate epitheliopathy and/or epithelial lesions	Corneal scrapes: culture and microscopy; PCR tests	1 mixed case of *Vermamoeba* and *Vahlkampfia*, 7 positive *Vermamoeba* cases (detected in isolation) (all CL wearers)	Propamidine and polyhexanide
Arnalich-Montiel et al. (2013) *Cornea* [24]	CS (Spain)	Analysing causative agent(s) of keratitis in a small cohort with suspected AK (5 F and 2 M patients)	Subepithelial changes, ulceration, ring infiltrates and stromal keratitis	Epitheliectomy and corneal scrapes: culture and microscopy; PCR tests	2 cases of mixed keratitis with *Acanthamoeba* and *Vahlkampfia* (both CL wearers)	Chlorhexidine, Propamidine, polyhexamethylene voriconazole and amniotic membrane transplant
Abedkhojasteh et al. (2013) *Iranian Journal of Parasitology* [9]	CR (Iran)	Analysing causative agent(s) of keratitis in a 22-year-old F soft CL wearer	Eye pain, photophobia, blurred vision, redness, tearing, foreign body sensation, opacity in epithelium and stroma	Culture and microscopy with CL, storage case and cleaning solutions; PCR tests	*Vermamoeba* detected (in isolation)	Polyhexamethylene
Hajialilo et al. (2015) *Iranian Journal of Parasitology* [10]	CS (Iran)	Analysing causative agent(s) of keratitis in a 23-year-old M and 21-year-old F soft CL wearers	Foreign body sensation, eye pain, photophobia, redness, tearing, burning, blurred vision and impaired vision	Culture and microscopy with CL and associating paraphernalia; PCR tests	1 mixed case of *Acanthamoeba* and *Vermamoeba*, 1 *Vermamoeba* positive case (in isolation)	Polyhexamethylene
Tolba et al. (2016) *PLOS Neglected Tropical Diseases* [25]	CR (Egypt)	Analysing causative agent(s) of keratitis in an Egyptian patient who incurred ocular trauma	Unexplained	Corneal scrapes: culture and microscopy; PCR tests	*Allovahlkampfia spelaea* detected (in isolation)	Unexplained
Pinna et al. (2017) *Cornea* [26]	CS (Italy)	Analysing causative agents of keratitis in a small cohort (43 M and F patients with suspected keratitis) 95% CL wearers	Corneal inflammation, keratoneuritis, epithelial defects and haze, pseudodendrites, ring infiltrates, inflammation and limbitis	Corneal scrapes: culture and microscopy; PCR tests	24 *Vermamoeba* positive cases detected (in isolation), 12 *Vahlkampfia* positive cases (in isolation), 3 mixed cases of *Vermamoeba* and *Vahlkampfia*	Polyhexamethylene (5 patients with advanced keratitis showed chronic visual impairment, likely requiring keratoplasty)

CR—Case Report, CS—Case Series, CL—contact lens(es), F—female, M—male, PCR—polymerase chain reaction, AK—*Acanthamoeba* keratitis.

**Table 2:** Summary of study characteristics.

**Study Type (n = 18)**	
CR	12
CS	6
**Publication Dates (n = 18)**	
1995–2009	11
2010–2019	7
**Location (n = 18)**	
UK	2
Ireland	1
USA	1
France	2
Japan	1
Germany	2
Spain	2
Turkey	1
Iran	3
Italy	2
Egypt	1
**Participants (n = 20)**	
Females ≤ 30 years old	5
Females > 30 years old	3
Males ≤ 30 years old	4
Males > 30 years old	1
CR unknown gender and age	1
^1^ Case series	6
**^2^** **CL history (n = 66)**	
CL wearer	61
Non-CL wearer	5

^1^ gender- and age-related information absent (excluding study by Hajialilo et al. [10]; this information has been recorded). ^2^ includes information from CRs and all CS, as CL history of amoebic keratitis positive cases was discussed.

**Table 3:** Amoebic keratitis causative agents and symptoms described across all studies.

**Amoebae sp. Detected**	**Percent of Cases (n = 66)**
*Vermamoeba*	53%
*Vahlkampfia*	23%
*Vermamoeba* and *Vahlkampfia*	7.5%
*Acanthamoeba* and *Vermamoeba*	6%
*Acanthamoeba* and *Vahlkampfia*	4.5%
*Vermamoeba* and *Vannella*	3%
*Paravahlkampfia*	1.5%
*Allovahlkampfia*	1.5%
**Symptoms**	**Percent of Studies (n = 18)**
Pain	61%
Inflammation, irritation and redness	61%
Stromal keratitis and ulcerations	56%
Epithelial erosion, defects, lesions and haze	50%
Ring infiltrates	50%
Impaired vision	44%
Photophobia	44%
Foreign body sensation	17%
Oedema	17%
Opacity	11%
Burning/stinging	6%
Discharge	6%
Keratoneuritis	6%
Pseudodendrites	6%

## Data Availability

Not applicable.
